# From Streptococcal Pharyngitis/Tonsillitis to Myocarditis: A Systematic Review

**DOI:** 10.3390/jcdd9060170

**Published:** 2022-05-25

**Authors:** Lukas Schmutzler, Moritz Mirna, Uta C. Hoppe, Michael Lichtenauer

**Affiliations:** Department of Internal Medicine II, Division of Cardiology, Paracelsus Medical University of Salzburg, 5020 Salzburg, Austria; u.hoppe@salk.at (U.C.H.); m.lichtenauer@salk.at (M.L.)

**Keywords:** cardiology, otolaryngology, myocarditis, streptococcus, pharyngitis, tonsillitis

## Abstract

(1) Background: Myocarditis following group A streptococcal pharyngitis and tonsillitis is a relatively rare medical condition. The aim of this systematic review was to identify specific ECG changes, laboratory parameters and signs, and symptoms associated with this disease. (2) Methods: A systematic literature review was performed in concordance with the current PRISMA guidelines, including the databases PubMed/MEDLINE, Web of Science, CDSR, CENTRAL, CCAs, EBM Reviews, and LILACS. Articles were included if they covered myocarditis after streptococcal pharyngitis/tonsillitis in humans. Exclusion criteria were rheumatic, autoimmune, or toxic myocarditis. (3) Results: Patients that developed myocarditis after group A streptococcal throat infection frequently presented with chest pain, elevated cardiac markers, and ST-segment elevations, making it a condition that shows more similarities to acute coronary syndrome than viral myocarditis. (4) Conclusions: Myocarditis after streptococcal pharyngitis and/or tonsillitis is a rather infrequently described disease; however, it is necessary to consider this condition when investigating streptococcal sore throat because it can be associated with severe adverse events for the individual patient.

## 1. Introduction

Both pharyngitis and tonsillitis are common diseases that typically emerge in winter and early spring. Even though pharyngitis is more often caused by viruses, *Streptococcus pyogenes*—i.e., group A streptococcus—is still responsible for approximately 15% to 30% of cases in children and 5–20% in adults [1,2].

The term “myocarditis” refers to an inflammation of the myocardium with various etiologies, amongst which viral pathogens are the most frequent. Other causes of this disease include bacteria, parasites, certain drugs, systemic diseases, and others [3,4]. While there is an increasing knowledge of myocarditis in general, relatively little is known about nonrheumatic myocarditis caused by group A streptococci. It was not until 1947 that Gore and Saphir first described patients who developed myocarditis after tonsillitis and pharyngitis without evidence of rheumatic fever [5].

Acute rheumatic fever (ARF) can potentially affect the entire heart (i.e., pericardium, epicardium, myocardium, and endocardium [6]) and although the exact pathophysiology of it is not understood to its full extent, molecular mimicry as well as autoimmunity definitely seem to play major roles in the disease mechanism [6,7]. Furthermore, ARF generally develops two to four weeks after the initial infection [7]. In contrast to ARF, nonrheumatic myocarditis following pharyngotonsillitis has a faster onset (usually a few days after streptococcal pharyngitis) and is thought to be caused by streptococcal toxins [8,9,10].

In this systematic review, we will first present the case of a young male who presented to our department with streptococcal tonsillitis and simultaneous myocarditis, then we will further review the available literature of this rather infrequent cardiac manifestation.

## 2. Case Presentation

**Figure 1:**
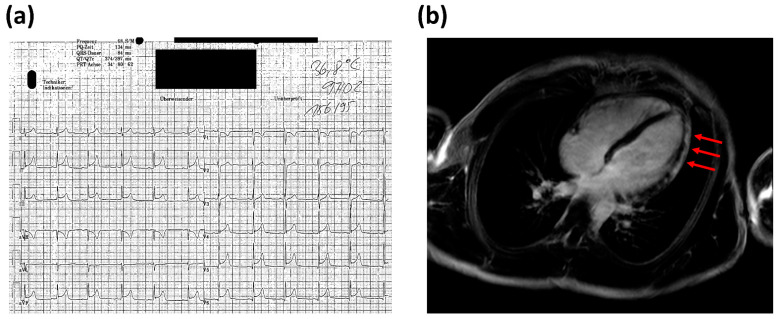
(**a**) ECG of the patient showed ST-segment elevations in leads II, III, aVF, and V4–V6; (**b**) in T1-weighted segments of cardiac MRI, subepicardial late enhancement in the area of the apex, as well as the anterolateral wall became apparent.

In 2012, a 19-year-old male presented to the emergency room of our university hospital with a primary complaint of acute chest pain. The day before, the patient was seen by his general practitioner because of high fever (up to 40 °C), sore throat, cough, and cervical lymphadenopathy. The general practitioner performed a throat swab, which was positive for *S. pyogenes*. Consequently, Penicillin V was administered, and the patient was discharged. However, because of severe chest pain, the patient presented to our emergency room the following day. The ECG showed ST-segment elevations in leads II, III, aVF, and V4–V6 (see Figure 1), the cardiac enzymes were significantly elevated (CK 858 U/L with a CK-MB fraction of 9.7%, hs-Troponin T 1042 ng/L (reference range: <14 ng/L)). The patient was admitted to the intensive care unit (ICU) for continuous monitoring. No abnormalities could be seen upon echocardiography; systolic left ventricular function was normal with no apparent regional wall motion abnormalities. Cardiac MRI showed subepicardial late enhancement in the area of the apex as well as the apical and midventricular anterolateral wall and the adjacent posterior wall (see Figure 1). Moreover, there was mild pericardial late enhancement suggestive of pericardial involvement. Furthermore, moderate apical and midventricular hypokinesia, as well as discrete dilatation of the LV (LVIDd 59 mm, LVEF 51%), became apparent upon MRI.

In 24 h Holter ECG, sinus rhythm with an increased number of PACs and PVCs was observed. However, there were no relevant ventricular arrhythmias. The patient was discharged from our hospital in a good general state of health one week after admission. A second MRI of the heart five months after discharge indicated a declining late enhancement of the myocardium and pericardium with emphasis on the apical and midventricular anterolateral wall. Another MRI was performed almost 3 years after initial presentation and showed more distinct areas of scarring compared to the second MRI.

## 3. Materials and Methods

The data of our case report were acquired as part of another retrospective study on patients admitted with a primary diagnosis of acute myocarditis. The study protocol of this study was reviewed and approved by the ethics committee of the state of Salzburg, Austria (EK Nr: 1181/2020) prior to data collection.

The systematic review was conducted according to the current Preferred Reporting Items for Systematic Reviews and Meta-Analyses (PRISMA) guidelines [11]. Literature search was performed by two independent reviewers (L.S. and M.M.) until March 2022, including the databases PubMed/MEDLINE, Cochrane Database of Systematic Reviews (CDSR), Cochrane Central Register of Controlled Trials (CENTRAL), Cochrane Clinical Answers (CCAs), Evidence-Based Medicine Reviews—Database of Abstracts of Reviews of Effects, Web of Science, and Latin American and Caribbean Health Sciences Literature (LILACS).

Search terms used were: “myocarditis AND (streptococc* OR tonsillitis OR pharyngitis) NOT rheumatic”. Studies were included if they met our predefined inclusion criteria, i.e., acute myocarditis in humans regardless of age following group A streptococcal pharyngitis or tonsillitis. Exclusion criteria were autoimmune/rheumatic origin of myocarditis, drug-induced toxic myocarditis, as well as previous streptococcal infection not affecting the pharynx or the tonsils and pharyngitis/tonsillitis caused by other groups of streptococci. Furthermore, abstracts, conference papers, and systematic reviews/meta-analyses were excluded from our review. Articles published either English or German were considered for inclusion, and there were no limits regarding the date of publication.

## 4. Results

### 4.1. Selected Studies

**Figure 2:**
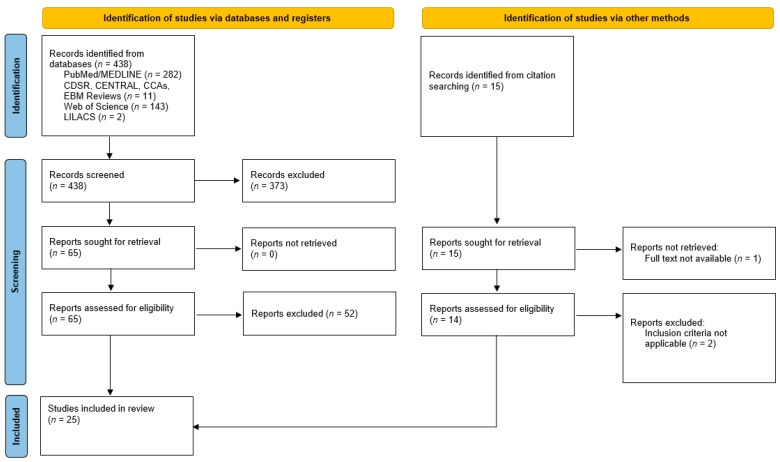
PRISMA flowchart of the study [11].

Figure 2 depicts the PRISMA flow diagram [11] of literature search and study selection of our systematic review. In total, 25 studies of 453 previously identified records were finally included in this review (see Table 1).

### 4.2. Patient Characteristics

Of all studies included in this review, there were 70 patients with myocarditis after streptococcal throat infection. 66 (94.3%) of these were male, whereas only 4 (5.7%) were female. The youngest patient was a six-year-old girl, while the oldest patient was a 62-year-old woman.

### 4.3. Clinical Characteristics

Chest pain was a typical finding in most patients (*n* = 54, 77.1%). Another prominent feature was dyspnea in eight patients (11.4%), while palpitations were described only in 1 patient (1.4%) in a study by Malnick et al., (2010) [24]. Other symptoms included “severe weakness”, diaphoresis, dizziness, nausea, epigastric discomfort, and vomiting. Moreover, two (2.9%) patients experienced syncope. Regarding the clinical signs, hypotension was mentioned in one (1.4%) subject and an irregular pulse (60 bpm) as well as a 2/6 systolic murmur heard at the base of the heart were observed in another patient. An additional finding was a pericardial friction rub, which was present in two patients [24,26]. In one patient, a loud second heart sound could be heard, while a loud S3, as well as an S4 heart sound were audible in another patient. Tachycardia, rales at the base of the lungs, and jugular vein distension were also observed in several patients. In addition to the aforementioned signs and symptoms, Gore and Saphir [5] reported five patients presenting with cyanosis, five experiencing dyspnea or orthopnea, three having cardiac arrythmias, one patient with Cheyne–Stokes respiration, seven patients with bronchopneumonia (of these, two interstitial), three with pulmonary edema, and six subjects with serous effusions. Furthermore, five patients of this study suffered an unexpected death. Another death was described in the study by Neagu et al., (2021) [12], where a six-year-old female died one day after the onset of sore throat, nausea, and vomiting. Therefore, a total of six patients (8.6%) died.

### 4.4. ECG Findings and Cardiac Markers

Interestingly, ST-segment elevations were observed in most cases (*n* = 33, 47.1%). Other abnormalities that could be seen on the ECG included T wave inversions, biphasic T waves, and other nonspecific ST-segment changes. Additionally, the ECG of one patient showed abnormal left axis deviation, as well as q-waves. Moreover, one patient had an alternating second-/third-degree atrioventricular block, and in one case, there was a non-sustained ventricular tachycardia. Cardiac enzymes were, if applicable, almost always elevated, except in the study by Mavrogeni et al. (2012) [10], where 9 out of 17 patients did not have elevated cardiac biomarkers, and in the study by Caraco et al., (1988) [32], in which one female had normal creatinine kinase values.

### 4.5. Imaging

#### 4.5.1. Transthoracic Echocardiography (TTE)

Of all patients included in the investigated studies, seven patients (10%) showed reduced left ventricular ejection fraction (<55%) at the time of admission, of whom four (57.1%) had heart failure with reduced ejection fraction (HFrEF; EF < 40%). Pericardial effusion was observed in five cases (7.1%) and was described as mild in all of them. Regional wall motion abnormalities were present in 15 subjects (21.4%), while a diffuse pattern of wall motion abnormalities was evident in two patients (2.9%). Interestingly, only one patient (1.4%) had moderate mitral regurgitation (MR), while all other patients had no relevant valvular pathologies upon the index TTE. The patient who had moderate insufficiency of the mitral valve was found to have accessory mitral valve tissue; however, this finding was supposedly not associated with acute myocarditis in this patient.

#### 4.5.2. Cardiac Magnetic Resonance Imaging (CMR)

Upon CMR, 25 patients (35.7%) showed late gadolinium enhancement, whereas early enhancement could be observed in 16 cases (22.9%). Furthermore, myocardial edema was present in 20 subjects (28.6%). The enhancement was described as subepicardial in 10 cases (14.3%) and as transmural in one individual (1.4%). One patient, however, had both subepicardial and transmural enhancement. The most frequently involved myocardial segment was the lateral wall (*n* = 17, 24.3%), followed by the septal (*n* = 11, 15.7%) and the apical (*n* = 11, 15.7%) regions. The least frequently affected segment was the anterior wall, with 1.4% (*n* = 1). The involved segment was not described in nine patients (12.9% and 28.1% of all 70 patients and of those 32 subjects where CMR was performed, respectively).

## 5. Discussion

In the Western world, viral pathogens are the most frequent cause for acute myocarditis [33]. Infrequently, however, myocarditis can also be the sequela of pharyngitis or tonsillitis caused by *S. pyogenes* (group A streptococcus) [20]. Despite the fact that this association has not been described very often, an increasing number of studies has identified group A streptococcal pharyngotonsillitis as the culprit of myocarditis (see Table 1).

*Clinical characteristics:* In the European Study of Epidemiology and Treatment of Cardiac Inflammatory Diseases (ESETCID) conducted in the year 2000, 71.7% of patients with suspected acute or chronic myocarditis presented with dyspnea, while 31.9% and 17.9% had chest pain and cardiac arrythmias, respectively [34]. In a study by Grün et al., chest pain was seen in 36.5%, and a combination of dyspnea on physical exercise, fatigue, and palpitations in 24.1% of patients diagnosed with viral myocarditis [35]. Interestingly, in our review, we found that chest pain was the most frequent symptom—observed in 77.1% of all patients—which is distinctively more frequent than in the papers described above. Moreover, dyspnea was a symptom in only 11.4% of patients in this systematic review. This low number, however, might be due to the fact that some articles did not focus on, and therefore did not mention, clinical characteristics or dyspnea in particular. In our review, only 1.4% of patients experienced palpitations.

*ECG findings and cardiac markers:* In a review by Buttà et al., the authors state that according to several studies, ST-segment elevations are present in 24% to 73% of patients with myocarditis. This difference in prevalence can be at least partly attributed to the transient ECG changes of myocarditis, meaning that patients who present at a later timepoint may have a normal ECG because their abnormalities could already have been resolved [36]. ST-segment elevations were the most encountered ECG abnormality in our review, seen in 57.9% of patients where abnormalities on ECG were described in more detail. Furthermore, T wave inversions were also seen, and this observation is consistent with the paper by Buttà et al., where the authors report that T wave inversions are the most frequently seen T wave changes in patients with a diagnosis of acute myocarditis [36]. Intriguingly, two patients in our review had biphasic T waves, which can sometimes be seen in ischemic changes, Wellens’ syndrome (especially in leads V2 and V3), and in pericarditis [37] but are not typical for myocarditis. In addition to T wave changes, nonspecific ST-segment changes have also been described, which are considered a typical ECG abnormality of viral myocarditis [4,35,38].

As outlined in Table 1, cardiac enzymes were elevated in most patients, with only 10 patients (14.3%) having normal levels of these biomarkers. However, a study by Smith et al. reported that CK-MB levels were elevated only in 6% and cardiac troponin I levels were elevated only in about a third of patients who were diagnosed with myocarditis [39]. This contrasts with our observations, although CK-MB and/or cardiac troponin I were not available for all patients. However, this might be an interesting finding suggesting that cardiac enzymes in streptococcal myocarditis following strep throat could be of greater importance than in myocarditis caused by other etiologies. On the other hand, a possible selection bias cannot certainly be excluded, thus warranting an observational trial addressing this aspect in the future.

*Transthoracic echocardiography (TTE):* Generally, the usefulness of echocardiography in myocarditis is limited, as the main reason for performing this diagnostic tool is to search for evidence of heart failure [4]. In our study, patients had a wide range of echocardiographic findings, including regional as well as global wall motion abnormalities, decreased LVEF, pericardial effusion, and mitral regurgitation (MR). These findings are comparable to those of a study by Angelini et al., where all previously mentioned echocardiographic features except MR have been described in affected patients [40]. 

*Cardiac Magnetic Resonance Imaging (CMR):* In CMR investigation, we found that late gadolinium enhancement was the most frequently observed finding which appears to be similar to viral myocarditis [41]. Moreover, a prospective study by Sanguineti et al. stated that CMR in patients with acute myocarditis showed subepicardial lesions most often (82.3%) and that the posterolateral wall was the most frequently affected segment (60%) [42]. This is in concordance with our results, in which the majority of patients had a subepicardial enhancement (14.3%) and lesions in the lateral wall (24.3%). Please note that many patients in our review did not undergo CMR and, therefore, the percentage of the aforementioned findings would be probably much higher if magnetic resonance imaging were performed on all patients.

The pathogenesis of nonrheumatic myocarditis following streptococcal pharyngitis or tonsillitis is still not clearly understood, and streptococcal toxins as well as cross-reactivity have been postulated as possible causative mechanisms [14,16,17,20]. However, the toxin theory seems to be more probable [22]. Interestingly, no bacteria could be identified in the histological samples of patients investigated in the paper by Gore and Saphir [5], which could corroborate the assumption that this type of myocarditis is toxin-mediated. The authors of the aforementioned study, which constitutes the earliest and most informative with regards to the autopsy findings identified in our search, mention that mononuclear cells, especially lymphocytes, were the most frequent inflammatory cells found upon histopathological examination. Furthermore, no abscesses could be identified in the histological samples of investigated patients [5]. In contrast to these observations, a recent article by Hiraiwa et al. described one patient with recurrent myocarditis in which both endomyocardial biopsy and autopsy showed an infiltration of neutrophils. In addition to that, micro-abscesses with bacteria were found at autopsy [43]. As this finding is the exact opposite of the observations by Gore and Saphir, future studies are required in order to achieve a better understanding of this rarely described pathology. Another interesting finding of the paper by Gore and Saphir was the presence of Aschoff cells [5], which are typical for rheumatic carditis [44]. Intriguingly, however, the authors observed that the Aschoff bodies did not have the particular perivascular location, which would be coherent with a rheumatic pathology [5]. Aside from that, no Aschoff bodies were reported in the case by Hiraiwa et al. [43].

## 6. Conclusions

In this systematic review, we observed that chest pain was a very typical symptom in patients who developed myocarditis after a group A streptococcal throat infection. Additionally, cardiac markers were elevated in the vast majority of patients, and ST-segment elevations could be observed frequently. These findings suggest that myocarditis after pharyngitis or tonsillitis caused by *S. pyogenes* might present with even more similarities (regarding clinical and diagnostic investigations) than viral myocarditis to acute coronary syndrome. Therefore, physicians—especially cardiologists, otorhinolaryngologists, and primary care doctors—should be aware of this potentially severe complication of group A streptococcal pharyngitis and tonsillitis, particularly if a patient develops chest pain simultaneously or a few days after strep throat.

## Figures and Tables

**Table 1:** Characteristics and principal findings of the studies included in this review.

Study (First Author, Publication Year)	Number of Patients (*n*)	Age (Years, Range)	Sex	ECG Findings	Cardiac Markers	Clinical Findings and Further Diagnostic Workup
Neagu, 2021 [12]	1	6	Female	N/A	N/A	Death within a day after the onset of symptoms (i.e., sore throat, nausea and vomiting) *Post-mortem histological features:* Diffuse severe myocarditis with involvement of pericardium and AV node
Kalpakos, 2021 [13]	1	33	Male	STE (posterolateral leads)	+(Troponin T, CK, transaminases)	Chest pain, epigastric discomfort and nausea *Echocardiography:* inferior-lateral hypokinesia *Cardiac MRI:* myocarditis (inferior-lateral segment of left ventricle)
Derbas, 2019 [14]	1	25	Male	STE (V2-V5)	+(Troponins (NT-pro-BNP))	Chest pain (pleuritic) as well as dyspnea *Echocardiography:* global hypokinesia, LVEF 37% *Cardiac MRI:* left ventricular dysfunction with edema in the apical and mid-anterior wall without late gadolinium enhancement
Müller, 2019 [15]	1	31	Male	N/A	+(Troponin T, pro-NTBNP)	Chest pain *Echocardiography:* regional wall motion abnormalities of the left ventricle (posteromedial and inferomedial akinesia); moderate mitral valve insufficiency; accessory mitral valve tissue (AMVT) (no clinical correlation between AMVT and myocarditis)
O’Brien, 2018 [16]	1	17	Male	STE (II, III, aVF, V4–V6)	+(Troponin, CK-MB)	Chest pain that worsened when lying down and during deep inspiration as well as additional dyspnea *Echocardiography:* LVEF 49% *Cardiac MRI:* transmural delayed enhancement of the mid-septum and apex
Silva, 2018 [17]	1	18	Male	*1st episode:* STE (I, II, aVL, V4–V6) *2nd episode:* STE (V3–V5), T wave inversion (I, aVL, V3–V4)	+(Troponin I)	Chest pain *Cardiac MRI:* subepicardial enhancement in the inferolateral wall *Additional information:* 2nd episode 2 weeks after resolution of initial symptoms
Pourmand, 2017 [18]	1	34	Male	STE (mild; inferior limb leads)	+(CK, CK-MB, Troponin)	Radiating chest pain (to back and left and right arm) *Echocardiography:* mild pericardial effusion
Sturmberger, 2016 [19]	1	26	Male	Biphasic T waves (II, III, aVF, V1–V4)	+(CK, CK-MB, high-sensitivity Troponin I)	Chest pain *Echocardiography:* normal findings *2D speckle tracking imaging:* decrease of systolic longitudinal strain of the posterior and lateral LV-wall segments *Cardiac MRI:* subepicardial delayed enhancement at lateral and posterior walls
Aguirre, 2015 [20]	1	43	Male	STE (I, II, aVF, V4–V6)	+(Troponin I, CK-MB)	Chest pain, epigastric pain *Cardiac MRI (17 day after discharge):* T2 signal intensity involving the subepicardial myocardium
Chikly, 2014 [21]	1	37	Male	STE (II, III, aVF)	+(hs-Troponin T, CPK)	Chest pain *Echocardiography:* akinesia of the inferior wall *Cardiac MRI:* T1 hyperintense areas in the inferolateral wall *Additional information:* This patient had a quite similar cardiac condition 5 years ago (also a few days after a group A streptococcal pharyngitis)
Chaudhuri, 2013 [22]	2	Mean 23.5 (18–29)	Male	STE in both patients	+(Troponin I)	Chest pain (patient 1 left-sided; patient 2 positional and precordial) *Echocardiography:* LVEF 48% and akinesia in the region of the apex in 1 patient and LVEF 52% and hypokinesia of the apex as well as mild pericardial effusion in the other one *Cardiac MRI (in one patient):* myocardial edema as well as subepicardial and intramural late gadolinium enhancement
Mavrogeni, 2012 [10]	17	Median 23 (18–29)	Male	Inverted T waves (V2–V6 and/or II, III, aVF in 15 patients) Nonspecific ST changes (in 2 patients)	+ in 8 patients (CK-MB and Troponin I) − in 9 patients	15 patients with severe chest pain, atypical chest discomfort experienced by 2 patients *Cardiac MRI:* 13 patients with normal LV function; impaired LV function in 4 patients (median LVEF 49.5%—significantly reduced EF compared to other patients); T2 enhancement in 16 patients; EGE values increased in 16 patients; LGE in 13 patients
Upadhyay, 2012 [23]	5	Mean 32.6 (22–47)	Male	STE (inferior: 2 patients; lateral: 1; inferolateral: 1; diffuse: 1)	+(Troponin T; in 1 patient N/A)	Chest pain
Malnick, 2010 [24]	4	Mean 30.5 (29–32)	Male	STE in 3 patients (I, aVL, V2–V6; inferior wall; minimal ST elevations in I and II); “pronounced T waves” in 1 patient (lateral leads)	+(in all patients Troponin, in one also AST, LDH, CPK)	Chest pain (2 radiating; in 1 patient to left arm, in other patient also scapular pain with radiation to left arm and shoulder); palpitations in one patient; pericardial friction rub in one patient; syncope in one patient
Mokabberi, 2010 [25]	8	Mean? (20–35)	7 Male, 1 Female	STE (5 anterolateral, 3 inferior)	+(CK in 7, CK-MB in 8, Troponin T in 8)	Chest pain (non-pleuritic) *Echocardiography:* regional wall motion abnormality in all patients; mitral regurgitation in 4 patients (trivial-to-mild) *Cardiac MRI (performed in 7 patients):* subepicardial LGE in all patients; LVEF slightly reduced in 4 patients
Talmon, 2009 [26]	2	Mean 48.5 (35–62)	1 Male, 1 Female	ST-T changes in both patients	+(Troponin I) in one patient	Pericardial rub in one patient and chest pain in the other patient
Khavandi, 2008 [27]	1	25	Male	STE (I, aVL)	+(Troponin I)	Radiating chest pain (to arms) *Cardiac catheterization:* apical hypokinesia (contrary to echocardiography which did not show any visible regional wall motion abnormality); elevated LVEDP (20 mmHg)
Kochar, 2008 [28]	1	18	Male	Left axis deviation; Q waves (III, aVF)	+(BNP and Troponin I)	Syncopal episode; worsening dyspnea during physical activity; hypotension; rales (base of the lungs); jugular vein distention *Echocardiography (initial):* globally hypokinetic LV; EF 20–25% *Echocardiography (few days later):* worsening of EF (10–15%); contrast throughout LV cavity (consistent with low flow state) *Echocardiography (2 weeks after discharge):* large and mobile homogenous echodensities (suggesting clots); resolution of contrast seen in previous echocardiography *Biopsy:* giant cell myocarditis
Talmon, 2008 [8]	2 (other 9 only suspected)	Mean 24.5 (17–32)	Male	Negative T waves in 1 patient; STE in other patient	+(CK, LDH, Troponin I)	Chest pain in both patients; loud second heart sound in 1 patient *Echocardiography:* mild pericardial effusion in 1 patient
Said, 1998 [29]	1	38	Male	STE (II, III, aVF, V5–V6); peaked T waves (precordial leads); non-sustained ventricular tachycardia	+(CK, CK-MB, AST, ALT, LDH)	Chest pain that worsened with respiration; dizziness, nausea and diaphoresis *Echocardiography:* slightly increased LVEDD (59 mm); diffuse hypokinesia; reduced LVEF (39%); mild mitral regurgitation; mild pericardial effusion
Gill, 1995 [30]	1	16	Male	STE (II, III, aVF); T wave inversions (V1–V2)	+(CK and LDH)	Radiating chest pain (to left arm) as well as nausea and vomiting *Left ventricular angiography:* myopathic left ventricle with normal coronary arteries; EF 25% *Echocardiography:* slightly enlarged left ventricle (with reduced EF) *Biopsy of right ventricle:* lymphocytic myopericarditis
Putterman, 1991 [31]	1	20	Male	STE (I, II, aVL, aVF, V4–V6); T wave inversions (V1–V3)	+(CK, CK-MB, AST, LDH)	Radiating chest pain (to left arm)
Karjalainen, 1989 [9]	3 (2 case reports and 1 prospective study)	Mean 20.5 (20–21) 3rd patient’s age N/A	Male	Patient 1: STE (I, II, aVL, V3–V6 + peaked T waves) Patient 2: STE (II, III, aVF, V3–V6) Patient 3: convex ST segment (I, V3–V6) with biphasic T waves; negative T waves (II, III, aVF)	+(CK and CK-MB) 3rd patient N/A	*Clinical characteristics:* Patient 1: left-sided chest pain (non-radiating) Patient 2: chest pain radiating to the left arm, nausea, loud S3 as well as an S4 heart sound Patient 3: did not experience any cardiac symptoms *Echocardiography:* Patient 1: LVEDD 58 mm, hypokinesia of the apex (anterior wall) Patient 2: LVEDD 49 mm, hypokinesia of the inferior wall of the LV, mild pericardial effusion *Other imaging:* Patient 1: positive myocardial scan (99mTc) Patient 2: moderate enlargement of the left heart as well as mild pulmonary venous congestion on chest X-ray
Caraco, 1988 [32]	1	38	Female	Alternating second-/third-degree AV block	−(CK normal and other cardiac markers N/A)	Irregular pulse (60 bpm); 2/6 systolic murmur at base of the heart
Gore, 1947 [5]	11	Mean 24 (18–33)	Male	Abnormal ECG in 1 patient	N/A	*Clinical characteristics:* cyanosis present in 5, dyspnea/orthopnea in 5, Cheyne–Stokes respiration in 1, cardiac arrhythmia/irregularity in 3, unexpected death in 5, bronchopneumonia in 7 (of these, 2 interstitial), serous effusions in 6, and pulmonary edema in 3 patients *Histology (in brackets estimated severity):* diffuse type in 5 cases (1 mild, 3 moderate, 1 marked), mixed type in 2 cases (2 moderate), and interstitial type in 4 patients (1 mild, 3 moderate)

Abbreviations: ECG: electrocardiography, N/A: not applicable, STE: ST-segment elevation, +: elevated, −: normal, CK: creatine kinase, MRI: magnetic resonance imaging, (NT-pro)BNP: (N-terminal pro-)B-type natriuretic peptide, (LV)EF: (left ventricular) ejection fraction, CPK: creatine phosphokinase, LV: left ventricle, AST: aspartate aminotransferase, LDH: lactate dehydrogenase, EGE: early gadolinium enhancement, LGE: late gadolinium enhancement, LVEDP: left ventricular end-diastolic pressure, ALT: alanine aminotransferase, LVEDD: left ventricular end-diastolic diameter, AV block: atrioventricular block, bpm: beats per minute.

## Data Availability

The data underlying this review will be shared by the corresponding author upon reasonable request.
